# Supplementary material for: The development and pilot testing of an ACP simulation-based communication-training program: Feasibility and acceptability
Source: PLoS One. 2021 Aug 24;16(8):e0254982. doi: 10.1371/journal.pone.0254982 (PMC8384223; doi:10.1371/journal.pone.0254982)
Supplement: S1 File — (PDF) [file pone.0254982.s001.pdf]

ACP 模擬情境
溝通訓練手冊

學員姓名:________________________

高雄醫學大學　護理系

目錄

內　　　　　　　　容	頁數
ACP 模擬情境溝通訓練工作坊簡介	2
ACP 模擬情境溝通訓練工作坊學員須知	3
第一週活動內容	4
第一週反思週記	5
第二週活動內容	6
第二週反思週記	7
第三週活動內容	8
使用 PREPARED model 的範例	9
情境案例 I	16
情境案例 II	17
情境案例 III	18
情境案例 IV	19
ACP 溝通訓練成效自我評量表	20
課前指定閱讀資料相關連結	21

「ACP 模擬情境溝通訓練」工作坊簡介

目的: 本工作坊旨在透過模擬情境溝通訓練，來增進護理人員與個案及家屬討論 ACP 的能力。訓練過程中，護理人員應用 PREPAERED model 溝通指引與不同案例的標準病人進行預立醫療自主計畫(ACP)溝通，期待個案及家屬能及早思考未來的醫療照護選擇，達到善終的目的。

本「ACP 模擬情境溝通訓練」工作坊，課程為期三週，每週進行一次，每次四小時，總共 12 小時。藉由此課程您將學習到:

- ACP 相關知識
- ACP 溝通技能

若您對本課程有任何問題，歡迎與研究人員陳瑞娥聯絡與討論

連絡電話: 0928999081

「ACP 模擬情境溝通訓練」工作坊學員須知

為使本工作坊能順利進行，達到模擬情境溝通訓練的目的，請學員能夠於:

- 課程活動開始前完成課程指定閱讀。
- 第一週與第二週課程結束後，分別書寫「反思週記」一篇。(詳見 P5.P7)
- 第二週與第三週課程一開始時，每位學員分享自己在上一週所寫的反思週記內容。
- 工作坊結束後三個月內，能嘗試在工作單位與病人討論 ACP，並試請病人勾選醫療照護意向量表 (https://hpcod.mohw.gov.tw/HospWeb/RWD/PageType/acp/acpa.aspx)；自己則填寫 ACP 溝通訓練成效自我評量表(P20)。

第一週

■ **學習目標:**

1.知識層面:學員能認識正確的 ACP 相關知識。

2.技術層面:學員能學習到討論 ACP 的溝通技能。

■ **活動設計:**以團體導向學習(Team Based Learning, TBL) 方式進行。

■ **活動內容:**

1.個人課前準備度測試

2.團體課程準備度測試

3.PREPARED Model 溝通指引介紹與範例說明

4.撰寫 Role play 劇本:由主持人提供案例情境,學員分組(3 人一組)進行 Role play 劇情撰寫,演出內容長度約 15 分鐘。

5. 每組依其撰寫的劇本進行 Role play。

6. 每組 Role play 結束後立即進行 Feedback:

(1) 人員: 角色扮演者、非角色扮演者(觀察學員)及主持人

(2) 內容: a.角色扮演互動過程中的感受或想法 b. 優點與缺點:應用溝通技能的表現(包含語言及非語言) c. 對角色扮演中護理師溝通技能的建議。

■ **家庭作業:**

➢ 書寫反思週記一篇

■ **下週活動準備:**

➢ 請攜帶溝通訓練工作坊手冊

➢ 上課前分享反思週記(每人 3-5 分鐘)

(分享重點:***在臨床情境應用時***遭遇到哪些困難? 如何克服或修正?)

➢ 各組組員自行分配第二週 Role play 演練的案例:

(3 位學員分配案例 I;另 3 位學員分配案例 II)

第一週反思週記

一、本週案例情境 Role play 劇情撰寫中，應用到哪些溝通技能?

二、Role play 演練後，透過自我反思、小組成員及主持人的 Feedback 過程中，學習到什麼?

三、請寫下在臨床情境應用過程中遭遇到哪些困難?(請敘述當下情境及反應)

四、若您未來在臨床工作上遇到類似的困境時，您覺得要如何修正會做得更好?

第二週

■ **學習目標:**

1. 知識層面:學員能正確應用 ACP 相關知識。
2. 技術層面:學員能熟練地應用 PREPARED Model 與標準病人討論 ACP。

■ **活動設計:** 以小組方式進行(6 人/組) Role Play。

■ **活動內容:**

1. 上課開始時，每位學員先分享自己在上一週所寫的反思週記內容。
2. 每位學員輪流應用 PREPARED Model 與標準病人進行案例 I 與案例 II 的角色扮演。
3. 每位學員 Role play 結束後立即進行 Feedback:
   (1) 人員:角色扮演者、觀察學員、標準病人及主持人
   (2) 內容: a.角色扮演互動過程中的感受或想法 b. 優點與缺點:應用溝通技能的表現(包含語言及非語言) c. 對角色扮演中護理師溝通技能的建議。
4. 綜合討論

■ **家庭作業:**

- ➢ 書寫反思週記一篇

■ **下週活動準備:**

- ➢ 請攜帶溝通訓練工作坊手冊
- ➢ 上課前分享反思週記(每人 3-5 分鐘)
  (分享重點:***在臨床情境應用時***遭遇到哪些困難? 如何克服或修正?)
- ➢ 各組組員自行配第三週 Role play 演練的案例:
  (3 位學員分配案例 III；另 3 位學員分配案例 IV)

第二週反思週記

一、本週的案例情境讓我在與標準病人互動的過程中，感到最困難的部分是什麼?

二、透過自我反思、小組成員、標準病人及主持人的 Feedback 過程中，學習到什麼?

二、本週的課程為我帶來什麼收穫或成長?請具體說明

四、請寫下在臨床情境應用過程中遭遇到什麼困難?(請敘述當下情境與反應)未來若遇到類似情境會要如何修正會做得更好?

第三週

■ **學習目標:**

1.知識層面:學員能正確應用 ACP 相關知識。

2.技術層面:學員能精熟 PREPARED Model 的應用，與標準病人討論 ACP。

■ **活動設計:** 以小組方式進行(6 人/組) Role Ply。

■ **活動內容:**

1.上課開始時，每位學員先分享自己在上一週所寫的反思週記內容。

2.每位學員輪流應用 PREPARED Model 與標準病人進行案例 III 與案例 IV 的角色扮演。

3. 每位學員 Role play 結束後立即進行 Feedback:

(1) 人員:角色扮演者、觀察學員、標準病人及主持人

(2) 內容: a.角色扮演互動過程中的感受或想法 b. 優點與缺點:應用溝通技能的表現(包含語言及非語言) c. 對角色扮演中護理師溝通技能的建議。

■ **家庭作業:**

工作坊結束後三個月內，能嘗試在工作單位與病人討論 ACP，並試請病人勾選醫療照護意向量表(https://hpcod.mohw.gov.tw/HospWeb/RWD/PageType/acp/acpa.aspx)；自己則填寫 ACP 溝通訓練成效自我評量表(P20)。

使用 PREPARED 模式的溝通範例

案例

(一) 病人資訊(Patient information)

姓名: 王○○	教育程度: 國小
性別: 女	宗教信仰: 民間信仰
年齡: 62	主要語言: 台語
婚姻: 已婚	主要診斷: DM. CKD
子女數: 一子	主要照顧者: 先生
職業: 家管	

(二) 情境敘述(Situation statement)

王太太罹患糖尿病已 10 年之久，由於未按時服藥，飲食也未節制以至於血糖控制不穩定，近期常出現夜尿情形，在上個月的定期回診檢查發現尿液檢查有蛋白尿、及血尿情形，經醫師轉診至腎臟科進一步檢查，診斷為早期腎臟疾病(early CKD)，醫師將其轉介給 CKD 衛教師進行腎臟疾病的相關衛教。**王太太進入衛教診間立即拉著衛教師的手說: 醫師說我有腎臟病會不會很嚴重?要不要洗腎....。**

(三)角色扮演

CKD 衛教師 (引導 ACP 討論)

個案 (DM 併 CKD 患者)

(四)應用 PREPARED 模式做為溝通指引，進行會談

■ 會談前的準備

-確認會談時間及地點(門診會談室)

-告知其他工作人員將進行重要會談切勿干擾，會談過程關手機避免他人干擾而中斷

【P 與個案討論前準備】

-確認自己已了解個案病情，準備好可與個案或家屬討論 ACP 議題

【R 確認自己已準備好】

已充分了解個案及家屬的文化和宗教信仰及對病情告知的偏好

【E 了解病人與照顧者對告知的偏好】

■ 會談開始

衛教師: 王女士您好，請坐(衛教師與個案面對面而座，與個案能有眼神接觸)

王女士: 您好(一邊點頭，一邊在衛教師的招呼下坐下)

衛教師: 今天有家人陪您一起回診嗎?(確認是否有家屬陪同)

王女士: 我先生今天有事去找朋友，我是自己搭公車過來的。

衛教師: 外面天氣很熱，辛苦您了! 最近感覺如何? (先話家常，先緩和病人心情，開始關心病人狀況)

王女士: 最近不知怎麼樣夜間會常常起來上廁所

衛教師: 一個晚上大概幾次?

王女士: 有時 2 次，有時 3 次啦!

衛教師: 這樣的情況持續多久?

王女士: 大概快一個多月

衛教師: 那對您的生活有影響嗎? ***【E 鼓勵提問與進一步討論】***

王女士: 睡不好啊!有時候起來迷迷糊糊的，有一次還撞到櫃子差點跌到，我先生也被我嚇一跳，有時候上完廁所後就很難再入睡翻來翻去，有時自己也不知道什麼時候睡著的。

衛教師: 您覺得自己身體狀況的改變可能是什麼原因? ***【E 鼓勵提問與進一步討論】***

王女士: 糖尿病都麼久了，以前是會有尿道感染，劉醫師開藥給我吃就好了。我上次回來看門診，劉醫師有再幫我抽血檢查，醫師看完報告有跟我說我的腎功能不太好，他說是因為我的糖尿病一直控制不好，所以傷害到腰子(腎臟)，我也不知道為什麼會變這樣。

衛教師: 這個結果讓您覺得很難接受? ***【A 關注情緒反應】***

王女士: 唉!我沒讀什麼冊(書)，不知道為什麼糖尿病又變成腰子病(腎臟病)。

衛教師: 你對你的疾病了解有多少？***【E 鼓勵提問與進一步討論】***

王女士: 我就是糖尿病，醫師有開藥給我吃，護理師也有教我要怎麼飲食控制，也教我要怎麼運動，只是有時會忘記吃藥，偶爾出去散步，飲食就跟家人吃一樣。

衛教師: 王女士我要跟您澄清不是糖尿病又變成腎臟病，是因為血糖控制不穩定所引起的腎病變，是糖尿病的合併症 ***【P 提供訊息】***。那您知道什麼是腰子病(腎臟病)嗎? ***【E 鼓勵提問與進一步討論】***

王女士: 醫師說他現在有開藥給我吃，可以減緩腰子(腎臟)功能變不好的速度，他說腰子(腎臟)功能如果惡化會變尿毒，可能要洗腰子(洗腎)。

衛教師: 王女士我跟您解釋一下腎臟病分為五期，前面三期是腎臟病早期，需要控制好血糖，加上飲食及藥物控制，每半年檢查一下腎功能；第四期是嚴重腎衰竭，可能會出現水腫、倦怠或高血壓等症狀，要限制鹽分攝取改善水腫，低蛋白飲食及限制高磷飲食預防腎骨病變，並積極配合醫師的治療；第五期是末期腎臟病，身體無法排除體內廢物和水分，可以選擇透析治療或做腎臟移植的準備***【P 提供訊息】***。這樣的說明您能了解嗎?有任何聽不懂的地方，您都可以隨時發問***【E 鼓勵提問與進一步討論】***。

王女士: 會變這麼嚴重喔!

衛教師: 依據你對自己疾病的認識，您的期待是什麼? ***【E 鼓勵提問與進一步討論】***

王女士: 我當然希望我的血糖可以控制穩定，腰子病(腎臟病)不要惡化，可以跟平常一樣過日子，不要後來變成糖尿病也沒控制好，有要洗腎那就糟糕了。

衛教師: 您現在最擔心的是什麼事? ***【E 鼓勵提問與進一步討論】***

王女士: 心情很亂..(沉默 2 分鐘)。我糖尿病也 10 幾年了，日子也像平常一樣過沒想到會變成腰子病(腎臟病)，這個能治好嗎?

衛教師: 我想每個人聽到這結果也會跟您一樣情緒很複雜***【A 關注情緒反應】***，您現在是處於早期腎臟病的階段，只要將血糖控制穩定，配合飲食跟醫師的藥物治療就能延緩腎功能惡化***【R 給予實際希望】***。

王女士: 衛教師您的意思是沒辦法治療好只能控制，那如果控制不好以後就要洗腰子(洗腎)了。

衛教師: 聽起來您好像很擔心萬一腎臟病控制不好要洗腎這件事**【A 關注情緒反應】**，要不要多說一點您的想法?**【E 鼓勵提問與進一步討論】**

王女士: 我的阿姨 85 歲洗腎 10 年了，以前我們家族都會相約一起去聚餐、旅遊，自從她洗腎後就只能待在家裡沒辦法跟我們出來吃喝玩樂，而且每個禮拜有三天都要兒子載她去洗腎，很麻煩小孩，也沒有生活品質。

衛教師: 聽起來您很擔心自己以後變成那樣，萬一有一天您的腎臟功能變差，需要洗腎時，您會有什麼打算? **【E 鼓勵提問與進一步討論】**

王女士: 我真的沒想過這件事，像這麼重要的決定我們都會提出來跟家人一起討論。

衛教師: 的確一般人面對這種狀況真的很難立刻做決定，下次要不要邀請家人一起來討論。

王女士: 好呀!我回去先跟家人講。

衛教師: 您曾經想過未來如果疾病發展不如預期或惡化，您有什麼想法?**【E 鼓勵提問與進一步討論】**

王女士: 我是有聽人家說過糖尿病如果沒控制好，有的人好像眼睛會越來越模糊，有的人可能傷口都不會好，有的腳會爛掉要截肢，有的好像會細菌感染很嚴重要急救。

衛教師: 那您知道急救的過程嗎? ***【E 鼓勵提問與進一步討論】***

王女士: 我不是很清楚啦!可是我看電視上都有演，就是醫生護士在病人的胸部上一直壓，還有給他(她)電擊，我看人都會燒焦了很可怕。

衛教師: 如果一個人的疾病已經不可治癒了，進入疾病末期，有一天發生呼吸及心跳停止時，您覺得要不要給予急救? ***【E 鼓勵提問與進一步討論】***

王女士: 我是覺得不要啦!人不必活的那麼痛苦，反正總有一天都會死掉，該走就走，不必強求，不只自己痛苦，也拖累家人。

衛教師: 目前政府有推動預立醫療自主計畫的政策，就是希望國人能思考自己面對生命盡頭時，對醫療照顧的想法 ***【P 提供訊息】*** 。

王女士: 是喔!沒聽說過，您可以跟我說嗎?

衛教師: 好的!我跟您解釋一下，預立醫療自主計畫的目的就是希望人在清醒時可以為自己未來失去決定能力時的醫療作決定，例如當自己疾病進入末期階段，也就是無法治癒了，自己可以填寫意願書，希望自己在臨終階段時不希望被急救，不使用維生醫療，或是要接受安寧療護等，這些意願會註記在健保卡上，萬一有一天您無法表達時，醫師可以遵從您清醒時決定的醫療意向，就不會進行插管急救等 ***【P 提供訊息】***。不知道這樣您能聽懂嗎? ***【E 鼓勵提問與進一步討論】***

王女士: 您的意思是說我們可以自己決定以後病沒辦法醫治的時候要不要被急救。

衛教師: 對!也就是我們現在就能為未來先做醫療照護的安排。不知您對預立

醫療自主計畫的看法如何?***【E 鼓勵提問與進一步討論】***

王女士:我覺得政府這個政策很好，我以前都不知道，這個就是人家說的安樂死?

衛教師:王女士預立醫療自主計畫和安樂死不同，簡單來說預立醫療自主計畫是保障那些醫師已經沒辦法治療好他(她)們病的末期病人不需接受無效醫療，譬如說插管、急救或接呼吸器等，來減輕病人的痛苦，達到善終的目的。而安樂死是醫師給予藥物提前結束病人生命，目前台灣醫界無法接受，在台灣也尚未立法***【P 提供訊息】***。

王女士: 我覺得政府應該要大力推廣，讓大家都知道。

衛教師: 王女士看來您很認同預立醫療自主計畫，我希望您下次約家人一起來，我再跟大家詳細解釋預立醫療自主計畫及五款意願及同意書。我總結一下今天會談的重點提到腎臟病的分期、 您對疾病的認識及期待、急救過程以及預立醫療自主計畫的概念。您還有什麼問題或疑問要提出來討論的? ***【E 鼓勵提問與進一步討論】***那今天的會談就此結束，麻煩您再跟家人確定一下可以來醫院討論的時間，大約一個小時左右，那您再跟我約時間好嗎? 那我們今天的會談就結束了，謝謝您。

會談後將會談內容記錄在病歷上。***【D)文件記錄】***

情境案例 I

(一) 情境敘述(Situation statement)

許先生 68 歲，高中畢業，喪偶，與兒子及媳婦同住， 5 年前因糖尿病導致腎病變引起腎衰竭，開始執行一週三天的血液透析治療，日常生活可自行料理，三天前他在家中突感左側肢體無力、口齒不清被家人送入急診，診斷為右側中大腦動脈阻塞而住院，住院期間由於喝水容易嗆到，予以插鼻胃管灌食，今天您是照顧他的護理要幫他量血壓。

(二) 學習目標 (Learning goals)

1. 能應用同理心處理個案的負面情緒
2. 能與個案討論拒絕人工營養及流體餵養的議題

(三) 角色扮演 (Role play)

護理師 (啟動 ACP 討論)

標準病人個案 (希望終止維持生命治療及人工營養及流體餵養)

情境案例 II

(一) 情境敘述(Situation statement)

李先生 58 歲，貨車司機，有糖尿病及高血壓病史約 10 年左右，未定期門診追蹤及服藥控制。3 年前開始洗腎，今天在洗腎過程中，鄰床病友突然休克被進行急救，他被那景象嚇住了，病友在急救後被送往加護病房繼續治療，您是照顧他的護理師過來幫他收針。

(二) 學習目標(Learning goals)

1. 能與個案討論未來可能面臨特定醫療情境時的照護過程
2. 能與個案說明病人自主權利法的相關內容

(三) 角色扮演

護理師(啟動 ACP 討論)

標準病人個案(擔心萬一成為植物人拖累家人)

情境案例 III

(一) 情境敘述(Situation statement)

馮女士 71 歲，國小畢業，可用國台語溝通，育有一子一女皆已各自成家，目前與先生同住，夫妻感情融洽，馮女士為虔誠的佛教徒。4 年前開始接受血液透析，本身為 B 肝帶原者，4 個月前因食慾不佳，皮膚黃疸就醫，發現肝腫瘤且有肺部轉移。今日她在血液透析過程中主動跟護理師說: 「***我頭暈不舒服***」。

(三) 學習目標 (Learning goals)

1. 能應用同理心處理個案的負面情緒
2. 能與個案討論撤除(終止)維持生命治療的選擇及後續照顧

(四) 角色扮演 (Role play)

護理師 (啟動 ACP 討論)

標準病人個案 (希望撤除/終止維持生命治療措施)

情境案例 IV

(一)情境敘述(Situation statement)

黃老先生 85 歲，有輕微失智，已洗腎 2 年多，一星期前在洗腎過程中出現低血壓休克被急救過，這二天又因感冒引起肺炎而入院治療。今天黃老先生血氧突然開始下降，意識逐漸模糊，血壓也有開始下降的情形。當護理師進入病房探視個案時，案女坐在床邊望著黃老先生表情凝重。

(三) 學習目標 (Learning goals)

1. 能與家屬解釋急救與不急救後的情況，並了解家屬所期望的治療結果
2. 能說明及協助家屬完成 DNR 文件的簽署

(四) 角色扮演 (Role play)

護理師 (啟動 DNR 討論)

標準病人案女 (希望爸爸接受維持生命治療措施)

ACP 模擬情境溝通訓練成效自我評量表

為了評量「ACP 模擬情境溝通訓練工作坊」的學習成效:

1. 請自我檢視、反思參加此工作坊後，您個人在溝通技能上有哪些改變或突破？

2. 此工作坊的哪些活動【例如：團體導向學習(TBL)、角色扮演後學員與講師的回饋、反思週記的書寫……等】增進你的溝通能力與自信程度。請**具體地舉例說明其理由**。

3. 相較於參加此次工作坊以前，這三週的 ACP 模擬情境溝通訓練課程對您在臨床上實際與病人討論 ACP 的幫助有多大？(以 0~10 分表示，請在下圖中圈選一個數字。)

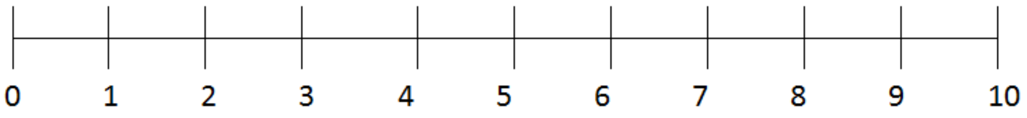

4. 您對 ACP 模擬情境溝通訓練課程的建議？

課前指定閱讀資料相關連結

1.台灣安寧照顧基金會官網

https://www.hospice.org.tw/care/law

- 安寧緩和醫療條例
- 意願書等五款表單介紹
- 病人自主權利法-本法
- 病人自主權利法-施行細則
- 預立醫療決定書
- ACP 預立醫療照護諮商說明工具-團隊版

2.衛生福利部國民健康署健康九九網站

https://health99.hpa.gov.tw/educZone/edu_detail.aspx?CatId=21853

- 預立醫療自主計畫手冊

預立醫療自主計畫參考資訊相關網站

- ◆ 衛生福利部 https://www.mohw.gov.tw/mp-1.html
- ◆ 台灣安寧照顧基金會 https://www.hospice.org.tw
- ◆ 病人自主研究中心 https://parc.tw/law/policy/article/262

發 行 人: 林秋菊

發行機構: 高雄醫學大學護理系

主　　編: 林秋菊

校　　稿: 大仁科技大學護理系　陳瑞娥

執行編輯: 大仁科技大學護理系　陳瑞娥

地　　址: 高雄市三民區十全一路 100 號

版　　次: 第一版

出版年月: 中華民國一 O 九年九月
