# Supplementary material for: The development and pilot testing of an ACP simulation-based communication-training program: Feasibility and acceptability
Source: PLoS One. 2021 Aug 24;16(8):e0254982. doi: 10.1371/journal.pone.0254982 (PMC8384223; doi:10.1371/journal.pone.0254982)
Supplement: Attachment — Submitted filename: notes for Authors.docx [file pone.0254982.s002.docx]

The topic is interesting. However the paper deals with the feasibility and acceptability of a training program carried out in 12 nurses only, which may be considered heuristic and the first step in a research project which is announced by the Authors.

24 ESRD: it is better to explain the abbreviation the first time it appears in your article.

Not only death …It could be better to explain that ESRD is a condition difficult to cope with, and distressing for the patients and their significant ones, with end-of –life decisions which can include situation in which patients are not able to speak for themselves, and so on…

29. “If advance care planning (ACP) can be discussed with patients and the ADs can be signed, patients may avoid the suffering of medical futility”

This statement is incorrect. ACP does not concern with medical futility.

32 disability

I suggest to use terms such as “severe impairment” and/or “new condition of life” and/or “treatments they would or would not be willing to endure”.

33 The phrase sounds wrong and should be reformulated to underline the prominent ethical value of ADC, that is autonomy.

31 I suggest to delete the term “medical” to consider care in an holistic approach aligned with patients’ preferences and values. It should be stressed tat ACP differs from general medical decision-making in being based on the person’s wishes for the time when their decisional capacity is impaired. As far as the family is concerned, it should be better to say that the family can be involved in the process, if the patient wishes... I understand that different cultural, ethical and legal issues may be important in how the family is involved in ACP.

44 I suggest to remove "including the scaffolding theory, the scenario simulation model, and the PREPARED model, as the basis of the ACP simulation-based communication training (ACP-SCT) program and adopted team-based learning (TBL) as the teaching method for program training”. It is confusing to me to have such terms anticipated and I can read about them easily in the next section “Theoretical framework “

70 Robinson et al

73-76 “ask them to preview the assigned reading materials before classes. In the second phase, learners will be asked to undertake the individual reading assurance test before classes so that lecturers can understand the learning outcomes before classes. After the individual test, the group readiness assurance test will be introduced to ensure the efficacy of group learning and to obtain the effect of real-time feedback. ask them to preview the assigned reading materials before classes “

I don’t understand… Please help me …

81 Eneanya et al

81-86 I suggest to move at the end of the introduction

112 Polit (typo)

150 The way in which recruitment was done is not clear. Probably as a consequence of this, I don’t understand the difference between recruitment and consent to participate. Moreover since the 12 nurses were enrolled by convenience sampling it is difficult to think that recruitment may be a way to measure feasibility

283 It should be correct to consider this paper as a first step within a research program. Is available the protocol of the “future definitive randomized clinical trial”? Is that trial protocol registered in a trail registry?

I think that educational program in palliative care and in ACP should involve all the health professionals at the same time (in this case both physician and nurse from hemodialysis room). Training programs could be more effective if carried out in multi-professional equipe dealing with with communication problems among heath professionals. In fact, a challenge in the ACP process may be the different approach of the health professionals involved in patients’ care for several reasons (different values, different skill, hierarchy, team flexibility and so on). Was this issue considered in the goal “improve communication skills of learners”? Did the ACP-STC program deal with team communication and factors within the health professional team that influence the success of the ACP process? How much concern was given to the item “D” of the PREPARED recommendations?
