# Supplementary material for: The development and pilot testing of an ACP simulation-based communication-training program: Feasibility and acceptability
Source: PLoS One. 2021 Aug 24;16(8):e0254982. doi: 10.1371/journal.pone.0254982 (PMC8384223; doi:10.1371/journal.pone.0254982)
Supplement: Attachment — Submitted filename: Response to reviewers.docx [file pone.0254982.s004.docx]

**Manuscript No:** PONE-D-20-39392

**Title: "** The development and pilot testing of an ACP simulation-based communication-training program: feasibility and acceptability**"**

| **Reviewer’s comments** | **Response to reviewers** |
| --- | --- |
| Reviewer #1: It would be important in the future to carry out a training and simulation path with different professionals with different training and backgrounds, to validate the central role of the team in advanced care planning. | Agreed. Added the reviewer’s comments to the limitations paragraph. (Page 15, lines 308-317) |
| Reviewer #3: Sorry, but I insist on the concept that educational program in ACP should involve all the health professionals at the same time who are involved in the patients' care. I would like that the Authors discuss this issue/limit of their study in the discussion. | Agreed. Thank you for the very important and specific suggestion. The limitations paragraph has been added to this study: (Page 15, lines 308-317)  ACP is a communication process in which various health professionals, such as physicians, nurse practitioners, social workers, and psychologists, discuss with patients and their families their personal preferences for future medical care in the face of loss of decision-making capacity. Accordingly, ACP is a person-centered, interdisciplinary, and collaborative approach to patient care that involves professionals of diverse backgrounds and areas of expertise. However, due to time, personnel, and resource constraints, only the nurses who are often the front-line caregivers were prioritized for training to test the feasibility and acceptability of this ACP-SCT program. It is recommended that future research could develop an ACP training program derived from our protocol but further includes a variety of healthcare professionals to validate the team's central role in ACP. |
